# Solute Carrier Family 35 Member F2 Regulates Cisplatin Resistance and Promotes Malignant Progression of Pancreatic Cancer by Regulating RNA Binding Motif Protein 14

**DOI:** 10.1155/2022/5091154

**Published:** 2022-05-27

**Authors:** Shuxian Zhang, Qingqing Li, Huixiao Yuan, Ling Ren, Xuyang Liang, Shouying Li, Shengxiang Lv, Hua Jiang

**Affiliations:** ^1^Department of Gastroenterology, Lianyungang Clinical College of Nanjing Medical University, Nanjing, Jiangsu, China; ^2^Department of Geriatrics, Shanghai Oriental Clinical Medical School of Nanjing Medical University, Shanghai, China; ^3^Department of Geriatrics, Shanghai East Hospital, Tongji University School of Medicine, Shanghai, China

## Abstract

We aimed to explore the role of Solute Carrier Family 35 Member F2 (SLC35F2) in pancreatic cancer (PCa) and to further study whether SLC35F2 regulates cisplatin resistance of PCa cells through the modulation of RNA binding motif protein 14 (RBM14) expression. SLC35F2 expression in 60 pairs of PCa tissues and adjacent ones was studied by RT-PCR analysis. Meanwhile, SLC35F2 expression levels in PCa cell lines were also evaluated by qPCR assay. In addition, SLC35F2 knockdown models were constructed in PCa cisplatin-resistant cells. Furthermore, we determined the interaction between SLC35F2 and RBM14 via luciferase assay. The findings of the present study demonstrated that SLC35F2 was significantly upregulated in PCa tissues. High level of SLC35F2 indicated higher incidence of metastasis and shorter survival rates. In vitro cell experiments revealed that knockdown of SLC35F2 suppressed cell invasion and metastasis capacity of cisplatin-resistant PCa cell lines PANC-1/DDP and CFPAC-1/DDP. It was also suggested that the key protein RBM14 in the SLC35F2 knockdown group was remarkably reduced. SLC35F2 can bind to RBM14 specifically. Overexpression of RBM14 partially reversed the effects of knockdown of SLC35F2 on the development of PCa. SLC35F2 expression in PCa tissues and cell lines is remarkably increased. In addition, it was also suggested that SLC35F2 may regulate cisplatin resistance of PCa cells through modulating RBM14 expression. In conclusion, it is conceivable from the study that SLC35F2 was remarkably upregulated in PCa and promoted the malignancy of PCa via regulating RBM14.

## 1. Introduction

According to the latest epidemiological statistics released by the American Cancer Society, there were an estimated 43,090 PCa-related deaths, including 22,300 in men and 20,790 in women. Meanwhile, this cancer has been the fourth leading cause of cancer-related death in men, accounting for 7% of all cancer deaths, second to lung and bronchial cancer (27%), colorectal cancer (9%), and prostate cancer (8%). Among female tumor-related cases, PCa accounts for 7% of deaths, also ranking fourth, after lung and bronchial cancer (25%), breast cancer (14%), and colorectal cancer (8%) [1–4]. However, for the early symptoms of PCa are not obvious, this disease was often found in advanced stage, leading to a very low five-year survival rate. At the time of diagnosis, less than 5% of tumor patients could be surgically removed [5–7].

Cisplatin is a first-generation platinum drug and a nonspecific cell cycle drug [8, 9]. The main targets of cisplatin are nucleophilic DNA, proteins, and RNA in cells [9, 10]. Currently, cisplatin is a broad-spectrum anticancer drug in the clinical treatment of tumors [10, 11]. However, patients often show some resistance in the treatment process, and the specific reasons still remain unclear [12]. One of the most important mechanisms of cisplatin resistance is the enhancement of DNA repair function and the cancellation of cisplatin's inhibitory effect on DNA repair [13, 14]. Effective DNA damage repair depends in part on structurally specific nuclease family members removing damaged groups or nucleotides and various intermediate DNA structures [14]. The SLC35F2 gene is located in the long arm of human chromosome 11, which is closely adjacent to it (11q22.3) by RAB39, a member of the Ras family of oncogenes [15]. In addition, SLC35F2 plays a key role in DNA replication and repair [16]. Therefore, this study intends to observe the effects of downregulation of SLC35F2 on the sensitivity to chemotherapy in PCa in vivo and in vitro, so as to lay a theoretical foundation for the clinical enhancement of the chemotherapy sensitivity of PCa and other malignant tumors.

## 2. Materials and Methods

### 2.1. PCa Samples

Human PCa samples were collected from 60 cases of surgically resected specimens of the pancreatic duct in Lianyungang Clinical College of Nanjing Medical University and Shanghai Oriental Clinical Medical School of Nanjing Medical University. All the samples had not received medical treatment and/or radiation treatment before surgery. The inclusion and exclusion criteria were according to a previous report [17]. Immediately after collection, the excised experimental specimens were immersed in an EP tube containing a preservation solution and stored in a −80°C refrigerator. All tissue samples used in the experiments were clearly diagnosed by two experienced pathologists. Signed written informed consent was obtained from the patients and/or guardians. This study was approved by the Ethics Committee of Lianyungang Clinical College of Nanjing Medical University.

### 2.2. Transfection

Lentivirus transfection was performed with sh-NC and sh-SLC35F2. Cells were collected for qPCR and western blotting analysis and cell function experiments. Cell transfection was performed according to the manufacturer's protocol step by step.

### 2.3. CCK8 Assay

Cells (PCa cell lines: AsPC-1, PANC-1, MIA PaCa-2, CFPAC-1, BxPC-3, and normal pancreatic ductal epithelial cell line HPNE) were prepared for the Cell Counting Kit-8 (CCK-8) assay according to the manufacturer's protocol.

### 2.4. Transwell Assay

Cell migration or invasion ability was measured using a 24-well plate cell according to instructions. Cells were then prepared into cell suspensions and seeded in the upper Transwell chamber (2.0 × 10^5^ cells/well). The migrated cells were counted and observed after being stained by crystal violet.

### 2.5. RT-PCR

After culture for 48 h, total RNA was extracted from transfected cells using TRIzol reagent (Beyotime, Shanghai, China) and then reverse-transcribed using the PrimeScript RT reagent kit (KeyGen company, Nanjing, China). The protocols of RT-PCR were according to a previous report [17]. Each sample was tested in triplicate wells and repeated three times. The primer sequences are shown in Table 1. Expression data were normalized by *β*-actin levels by the 2−ΔΔCt method.

### 2.6. Western Blotting Assay

Transfected cells were collected after 72 h of culture, and proteins were extracted for quantitative detection with the total protein concentration determined using the BCA method. Immunoblotting was carried out using primary rabbit polyclonal anti-SLC35F2 (dilution: 1 : 500; Cat. no.: ab 222854) and rabbit polyclonal anti-RBM14 (dilution: 1 : 500; Cat. no.: ab70636) with GAPDH (dilution: 1 : 500, Cat. no.: ab37168) as internal control, followed by incubation with the secondary goat anti-rabbit (HRP) IgG antibody (dilution: 1/2000; Cat. no.: ab6721). All the antibodies were purchased from Abcam (Cambridge, MA, USA). The semiquantitative analysis of the protein expressions was determined using alpha SP image software.

### 2.7. Luciferase Reporting Assay

PCa cells in good condition were used for luciferase assay. The relative fluorescence value was then detected by a photometer for comparison.

### 2.8. Statistical Analysis

SPSS 22.0 statistical software was used for data analyses. The relationship between SLC35F2 and clinicopathological characteristics was analyzed by *χ*^2^ test. *P* < 0.05 was considered to be statistically significant.

## 3. Results

### 3.1. SLC35F2 Was Highly Expressed in PCa

**Figure 1:**
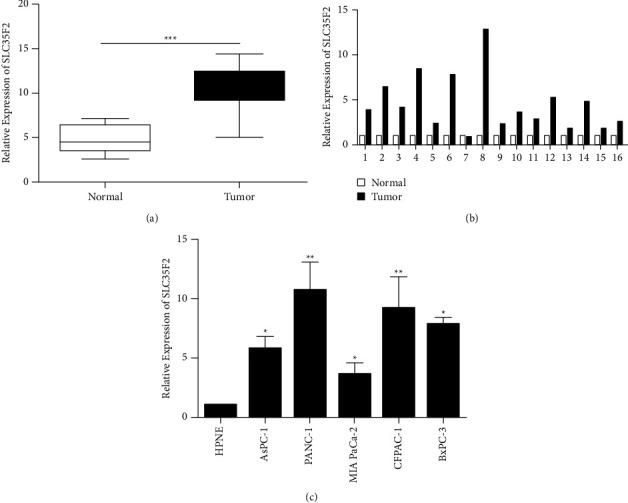
SLC35F2 is highly expressed in pancreatic cancer tissues and cell lines. (a, b) qRT-PCR detection of SLC35F2 expression in pancreatic cancer tumor tissue and nontumor tissue adjacent to the tumor; (c) qRT-PCR detection of SLC35F2 expression level in pancreatic cancer cell lines; Data are mean ± SD, ^*∗*^*P* < 0.05, ^*∗∗*^*P* < 0.01, and ^*∗∗∗*^*P* < 0.001.

qPCR detected SLC35F2 in 60 PCa samples and their adjacent tissues and PCa cell lines. Figures 1(a) and 1(b) show that tumor samples contained a significantly higher expression of SLC35F2 (Figures 1(a) and 1(b)). Meanwhile, SLC35F2 was markedly highly expressed in PCa cells (Figure 1(c)). Additionally, prognosis analysis revealed that high level of SLC35F2 indicated poor prognosis in patients with PCa (*P* < 0.05).

### 3.2. SLC35F2 Contributed to Cisplatin Resistance in PCa

**Figure 2:**
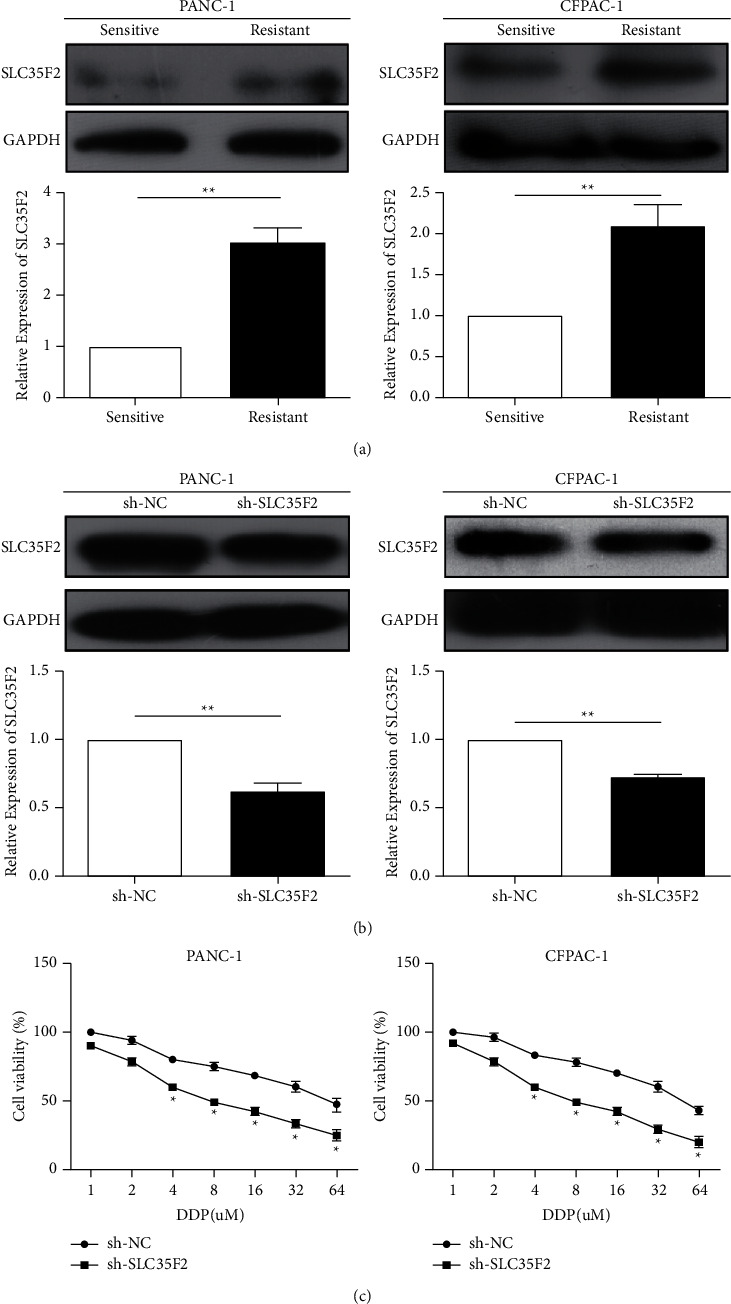
SLC35F2 increases the cisplatin resistance of pancreatic cancer cells. (a) qRT-PCR and western blotting detected SLC35F2 expression in cisplatin-resistant pancreatic cancer patients; (b) qRT-PCR and western blotting detected the expression level of SLC35F2 in pancreatic cancer cell lines PANC-1 and CFPAC-1 after transfection of the SLC35F2 knockdown vector; (c) CCK-8 cell proliferation experiments detected cell viability after transfection of SLC35F2 knockdown vector in pancreatic cancer cell lines PANC-1 and CFPAC-1. Data are mean ± SD, ^*∗*^*P* < 0.05, ^*∗∗*^*P* < 0.01.

Subsequently, qPCR and western blotting detected an increased expression of SLC35F2 in cisplatin-resistant PCa patients (Figure 2(a)). To further examine the impacts of SLC35F2 on the cisplatin resistance, SLC35F2 knockdown vectors were constructed in cisplatin-resistant PCa cells (Figure 2(b)). As a result, it was found that knockdown of SLC35F2 markedly attenuated the viability of the PCa cells (Figure 2(c)).

### 3.3. Knockdown of SLC35F2 Inhibited Cell Proliferation and Metastasis

**Figure 3:**
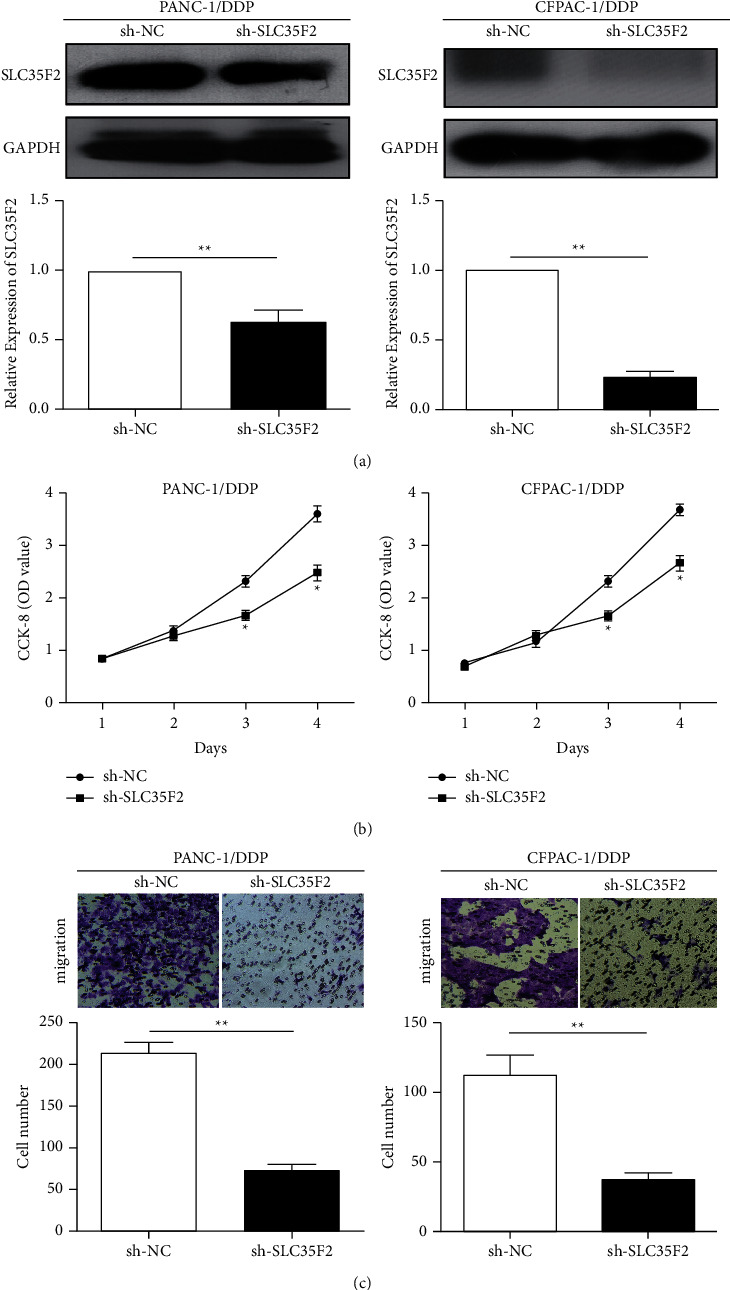
Silencing SLC35F2 inhibited pancreatic cancer cell proliferation and metastasis. (a) qRT-PCR and western blotting verified the interference efficiency after transfection of SLC35F2 knockdown vectors in pancreatic cancer cisplatin-resistant PANC-1/DDP and CFPAC-1/DDP cell lines; (b) CCK-8 assay detected the proliferation rate of pancreatic cancer cells after transfection of the SLC35F2 knockdown vector in PANC-1/DDP and CFPAC-1/DDP cell lines; (c) Transwell invasion assay detected the invasion of pancreatic cancer cells after transfection of the SLC35F2 knockdown vector in PANC-1/DDP and CFPAC-1/DDP cell lines. Data are mean ± SD, ^*∗*^*P* < 0.05.

Furthermore, to assess the influence of SLC35F2 on the proliferative ability of PCa cells, we constructed an SLC35F2 knockdown model in PCa cisplatin-resistant cells (Figure 3(a)). Subsequently, results showed that inhibition of SLC35F2 significantly attenuated the proliferative rate and invasiveness (Figures 3(b) and 3(c)).

### 3.4. RBM14 Was Lowly Expressed in PCa

**Figure 4:**
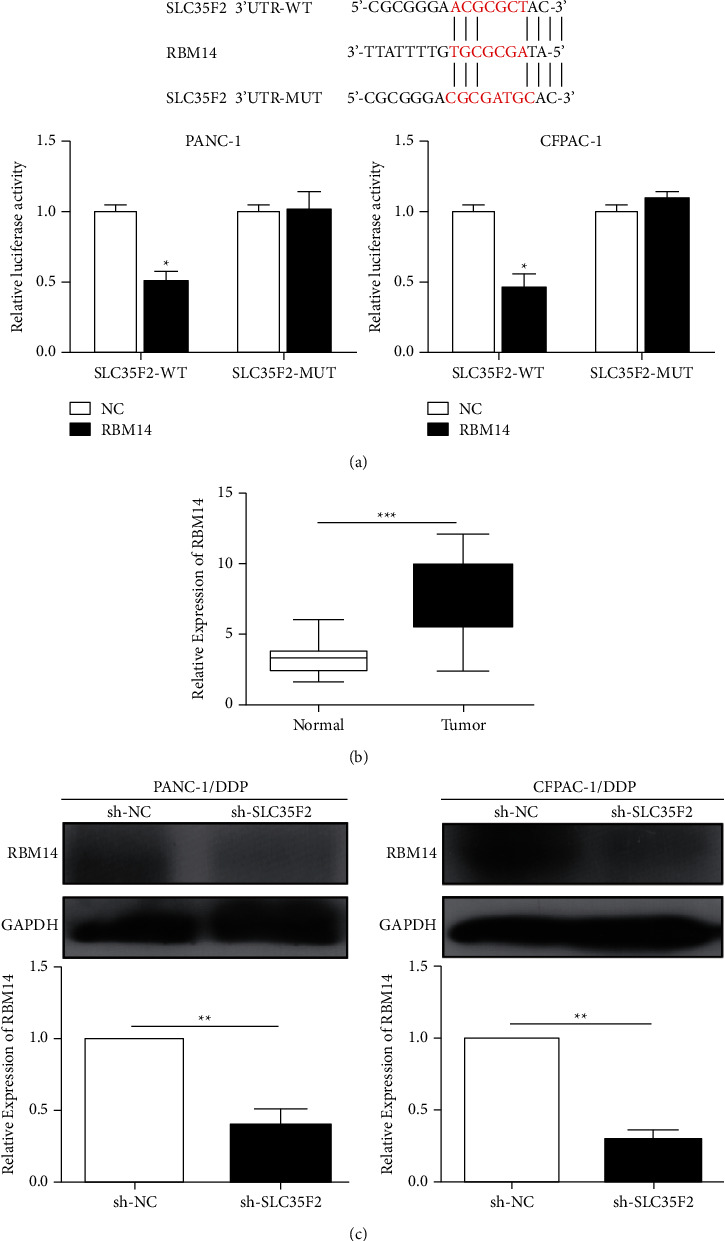
SLC35F2 can target RBM14. (a) Dual-luciferase reporter gene was used to verify the direct targeting of SLC35F2 to RBM14; (b) qRT-PCR detection of RBM14 expression differences in pancreatic cancer tumor tissue and adjacent tissues; (c) qRT-PCR and western blotting verified the expression level of RBM14 after downregulation of SLC35F2 in pancreatic cancer cisplatin-resistant PANC-1/DDP and CFPAC-1/DDP cell lines. Data are mean ± SD, ^*∗*^*P* < 0.05, ^*∗∗*^*P* < 0.01, ^*∗∗∗*^*P* < 0.001.

Bioinformatics research revealed that there may be some connection between SLC35F2 and RBM14 expression in PCa. Subsequently, SLC35F2 and RBM14 were cotransfected into PANC-1 and CFPAC-1 cell lines, and luciferase assay verified that SLC35F2 can directly bind to RBM14 (Figure 4(a)). In comparison with adjacent tissues, tumor tissue samples showed an increased expression of RBM14 (Figure 4(b)), which was positively correlated with RBM14 expression. Meanwhile, in cisplatin-resistant PCa cell lines PANC-1/DDP and CFPAC-1/DDP, knockdown of SLC35F2 remarkably reduced the protein expression of RBM14 (Figure 4(c)).

### 3.5. RBM14 Modulated SLC35F2 Expression

**Figure 5:**
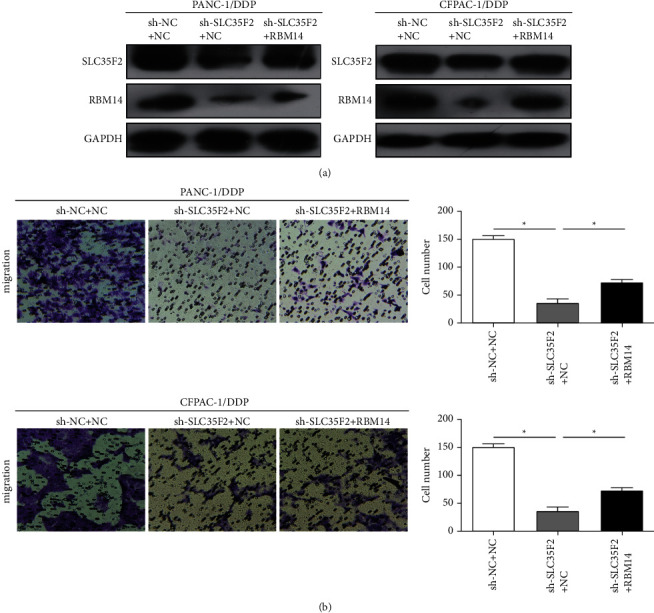
SLC35F2 regulates the expression of RBM14 in pancreatic cancer cells. (a) Western blotting was used to detect the expression levels of SLC35F2 and RBM14 after cotransfection of the SLC35F2 knockdown vector and RBM14 overexpression vector in pancreatic cancer cisplatin-resistant PANC-1/DDP and CFPAC-1/DDP cell lines; (b) Transwell invasion experiments examined the invasion of pancreatic cancer cisplatin-resistant PANC-1/DDP and CFPAC-1/DDP cell lines after cotransfection of SLC35F2 knockdown vector and RBM14 overexpression vector. Data are mean ± SD, ^*∗*^*P* < 0.05.

In order to investigate the association of SLC35F2 with RBM14, we overexpressed RBM14 in cisplatin-resistant PCa cell lines that silenced SLC35F2 and confirmed the transfection efficiency by western blotting (Figure 5(a)). Transwell assay demonstrated that overexpression of RBM14 reversed the effects of SLC35F2 downregulation on the invasive ability of PCa cells (Figure 5(b)).

## 4. Discussion

Currently, it is estimated that PCa will develop into the second leading cause of cancer-related mortality by 2020 [1–4]. Due to the lack of accurate early diagnosis methods and effective preventive measures, this cancer is usually diagnosed at advanced stages and shows a very poor response to chemotherapy drugs (including cisplatin) [6–8]. Recently, a variety of roles of SLC35F2 in cancers have been reported in different kinds of tumors [18–20]. Chen et al. demonstrated that SLC35F2 can promote malignant progression and is a potential therapeutic target in bladder cancer [18]. Li et al. showed that RNA interference-mediated downregulation of SLC35F2 expression by lentiviral vector can attenuate the proliferation, migration, and invasion of lung cancer cells [19]. Bu et al. found that highly expressed SLC35F2 was associated with non-small-cell lung cancer pathological staging and thus might be able to predict the prognosis of patients with NSCLC [20]. However, little evidence is available regarding the role of SLC35F2 in PCa.

In this study, we detected the SLC35F2 expression in 60 pairs of PCa patients' tumor tissues and adjacent tissues and found that it was remarkably upregulated in tumors and positively related to poor prognosis, indicating that SLC35F2 might be a cancer-promoting gene in PCa. In addition, to further understand the function of SLC35F2 in the formation and development of PCa, we knocked down SLC35F2 expression and found that downregulation of SLC35F2 increased the sensitivity of PCa to cisplatin and thus inhibited the proliferation and metastasis of PCa cells, indicating that SLC35F2 may inhibit the cisplatin sensitivity of PCa cells.

Bioinformatics analysis suggested that SLC35F2 could regulate RBM14 and thus affect cisplatin sensitivity, proliferation, and migration ability of PCa cells. We then confirmed the binding relationship and the positive regulation between SLC35F2 and RBM14 by a luciferase reporter gene assay and western blot. We subsequently found that overexpression of RBM14 significantly reversed the inhibitory effects of silencing SLC35F2 on PCa cell invasion, suggesting that SLC35F2 may regulate cisplatin resistance of PCa cells by modulating RBM14. Further understanding of the biological effects of SLC35F2 and RBM14 genes and their role in the development of tumors will be more helpful for the diagnosis, treatment, and prognosis evaluation of tumors.

## 5. Conclusion

In summary, SLC35F2 was remarkably upregulated in PCa, which was relevant to worse prognosis. Additionally, SLC35F2 may regulate cisplatin resistance of PCa cells and promote the malignancy of PCa via regulating RBM14.

## Figures and Tables

**Table 1:** Primer sequence.

Gene		Primer sequence
SLC35F2	Forward	5′-ATCCAGTGCGTGTCGTG-3′
	Reverse	5′-TGCTTGGAATGTAAGGAAG-3′
RBM14	Forward	5′-TCTCGGGGTGATCGACAAG-3′
	Reverse	5′-CCCTTTGTTCATTCGTTCCT-3′
*β*-Actin	Forward	5′-CCTGGCACCCAGCACAAT-3′
	Reverse	5′-GCTGATCCACATCTGCTGGAA-3′

## Data Availability

The datasets used and analyzed during the current study are available from the corresponding author on reasonable request.
